# The Evidence Informing Hypoglycaemia Treatment Thresholds in Asymptomatic, Term, Well Newborns: A Review of Literature and Critical Appraisal of Methodological Challenges

**DOI:** 10.1177/08903344261426051

**Published:** 2026-04-13

**Authors:** Kylie Hodges, Samantha Trenaman, Mary Bushell, Maryam Bazargan, Marjorie Atchan

**Affiliations:** 1 University of Canberra, ACT Australia; 2 NSW Health, NSW, Australia

**Keywords:** blood glucose, blood sugar level, hypoglycaemia, lactation, narrative review, neonatal, newborn, treatment thresholds

## Abstract

**Background:**

There is worldwide inconsistency in blood glucose level treatment thresholds for hypoglycaemia in healthy, term asymptomatic newborns. Differing testing policies and broader inclusion criteria from maternal conditions have increased the incidence of testing. The use of human milk substitutes to treat hypoglycaemia may adversely influence the establishment of breastfeeding and early bonding between mother and baby.

**Research Aim:**

To determine if current research informs a high-quality evidence-based therapeutic treatment threshold for hypoglycaemia in well, asymptomatic newborns in the first 24 hours of life.

**Methods:**

A comprehensive review accessing CINAHL, Medline, Scopus, Web of Science, Cochrane, and CENTRAL databases was performed. Original English-language quantitative studies reporting hypoglycemic treatment thresholds, and their neurodevelopmental outcomes for over 11,000 newborns, were appraised for quality and validity using independent screening and Crowe’s Quality Appraisal Tool.

**Results:**

Thirteen papers were included with outcomes grouped into two categorical definitions of hypoglycaemia (statistical and neurodevelopmental). Nine of the included studies were assessed as producing low quality evidence, with a moderate to high risk of bias, unreliable methodology, and smaller sample sizes. One study provided high quality evidence, with a sample size of > 1000 newborns, that a blood glucose threshold of 2.09 mmol/L was a potentially safe treatment threshold.

**Conclusion:**

Published high quality research is lacking to inform a therapeutic treatment threshold for hypoglycaemia in well, term asymptomatic newborns in the first 24 hours. Current treatment may interfere with breastfeeding and bonding. Rigorous research is required to establish a safe threshold and inform future practice.

## Background

Hypoglycaemia is a condition that occurs when blood glucose levels (BGLs) drop below a safe level to function optimally (4 mmol/L in adults; Diabetes Australia, 2022). A hypoglycaemic event can cause the brain to be starved of energy, potentially leading to seizures, loss of consciousness, and death (Diabetes Australia, 2022). Adequate stores and access to glucose from birth is essential to facilitate a newborn’s transition to extrauterine life in the first 24 hours. A proven metabolic compensatory physiological mechanism allows neonatal BGLs to initially drop to lower levels from those at birth (nadir) until natural correction occurs at approximately 3 to 4 hours of life, when their endocrine system regulates BGLs to within a normal range (Blackburn, 2017). Medical literature reports that hypoglycaemic episodes in newborns can lead to neurodevelopmental injury (Cornblath et al., 2000). A consensus on the lowest BGL to accept in newborns, and how to treat a hypoglycaemic episode, is essential to ensure neonatal safety for those at-risk newborns (Cornblath et al., 2000). Early BGL testing and intervention during the neonatal nadir period may also interfere with the establishment of normal glucose homeostasis (Adamkin & Committee on Fetus and Newborn, 2011).

**Key Messages**

- Consensus is lacking on a hypoglycaemia treatment threshold for neonates that balances potential health benefit with burden. The most commonly used hypoglycaemia threshold is 2.6 mmol/L, with a range of 2.0 mmol/L to 2.6 mmol/ reported.
- Low quality studies using inconsistent criteria create disparity in management and potential over-treatment.
- Over-treatment negatively affects the normal newborn’s extrauterine adaptation and may disrupt the establishment of successful breastfeeding.
- Further research is required to inform future practice

The monitoring of newborn BGLs is driven by symptoms or pre-determined risk factors (Adamkin & Committee on Fetus and Newborn, 2011). Theoretical risk factors in otherwise well newborns includes gestational and pre-existing maternal diabetes, maternal use of some medications in pregnancy such as beta blockers and valproic acid (valproate), prematurity, small for gestational age (SGA) or large for gestational age (LGA), and feeding issues (Adamkin & Committee on Fetus and Newborn, 2011).

The incidence of gestational diabetes and maternal co-morbidities in Australia has doubled over the last decade (Chappe, 2020; Diabetes Australia, 2022), leading to a doubled incidence of risk factors for testing in newborns (Mukhopadhyay et al., 2020). Despite the increase in monitoring, the definition and treatment threshold for hypoglycaemia is variable and confusing (Adamkin & Committee on Fetus and Newborn, 2011; Alsweiler et al., 2007; Cornblath et al., 2000; Dixon et al., 2017; Narasimhan et al., 2021).

Current hypoglycaemia clinical practice guidelines recommend that newborns who experience a hypoglycaemic event are treated with dextrose gel and a weight-based feeding schedule, or an intravenous dextrose infusion (Cordero et al., 2018), to increase the BGL to within the euglycemic range. The feeding schedule is in excess of the supply of colostrum that would normally be expected in the first 24 hours for most women (Kent et al., 2012), with commercial milk formulae being recommended as a supplementary feeding source (McKinlay et al., 2015).

A large body of evidence shows that any use of commercial milk formula interferes with the establishment of breastfeeding (Dalsgaard et al., 2019; McCoy & Heggie, 2020; Mukhopadhyay et al., 2020). The interventions used to manage BGLs, such as commercial milk formula and intravenous or oral feeds via nasogastric tubes, interfere with bonding and breastfeeding establishment, which negatively influence breastfeeding success rates (Dalsgaard et al., 2019; McCoy & Heggie, 2020; Rees et al., 2021; Saginur et al., 2023; Sivarajan et al., 2021; Wight & Academy of Breastfeeding Medicine, 2021; World Health Organization [WHO], 2003) and potentiate adverse health outcomes for the mother and newborn.

The use of commercial milk formulae alters the newborn’s gut microbiome (Forbes et al., 2018), increasing their risk of developing infections, such as otitis media, gastroenteritis, and pneumonia. The risk of childhood asthma, obesity, diabetes mellitus (Types 1 and 2), leukaemia, and sudden infant death syndrome are also increased (Gale et al., 2012; Stuebe, 2009).

Newborn hypoglycaemia treatment policies and protocols from around the world recommend commencing treatment of BGLs from as high as 3.3 mmol/L (Jesitus et al., 2016) to as low as 1.4 mmol/L (Jesitus et al., 2016; Wight & Academy of Breastfeeding Medicine, 2021) and different testing timelines. This inconsistency can also be seen in specific country settings, such as Australia, with clinical policies using thresholds from 1.5 mmol/L (Narasimhan et al., 2021) to 2.6 mmol/L being used (ACT Health, 2021; Harris et al., 2014; New South Wales Government, 2021; South Australian Maternity Neonatal & Gynaecology Community of Practice (SA), 2020; The Royal Children’s Hospital Melbourne, 2019), and thresholds in use for different times (i.e. lower thresholds for less than 4 hours of life (South Australian Maternity Neonatal & Gynaecology Community of Practice). It is estimated (Harris et al., 2014) that three quarters of current Australian policies and procedures for newborn screening of hypoglycaemia continue to utilise seminal research conducted in the 1980s (Koh et al., 1988) to determine treatment thresholds, which may not be clinically relevant.

Internationally, health professionals acknowledge that clinical guidelines are inconsistent and lack quality evidence (De Angelis et al., 2021; Rozance & Hay, 2016; United Nations Children’s Fund [UNICEF] – UK, 2013; Wight & Academy of Breastfeeding Medicine, 2021). With the growing incidence of risk factors leading to an increased rate of testing for hypoglycaemia in newborns (Diabetes Australia, 2022; Harris et al., 2020), it is essential that safe hypoglycaemic treatment thresholds for newborns are established. This will ensure that practice is evidence-based and standardised, and that newborns are not subject to unnecessary interventions (Sivarajan et al., 2021) given the knowledge of potential adverse health outcomes.

Best quality evidence is expected in current healthcare practice. The aim of this review is to determine if high-quality evidence currently informs the recommendations to commence the treatment of neonatal hypoglycaemia in the well, term newborn who may have (maternal) risk factors but are asymptomatic in the first 24 hours of life.

## Methods

### Design

This narrative review was not registered prior to commencement. Rodgers and colleagues’ (Popay et al., 2006) framework for narrative synthesis was utilised to provide the highest level of evidence to inform practice. The high degree of heterogeneity in the data, and discrepancies in the quality of research with no direct compatibility, led to incompatibility with a meta-analysis.

This review utilised the four universally accepted methods for defining neonatal hypoglycaemia as proposed by the WHO (1997) and Cornblath and colleagues’ work (Cornblath et al., 2000): statistical (or epidemiological), neurodevelopmental, neurophysiological, and endocrine. This review did not find any studies that utilised the endocrine definition. One study addressed the neurophysiological definition; however, it was excluded after CCAT appraisal (Koh et al., 1988). Our findings have been categorised according to the two remaining applicable categories: statistical and neurodevelopmental.

### Sample

Peer reviewed literature, published in English, that reports original studies (including randomised controlled trials (RCTs), quasi RCTs, cohort and observational studies) on treatment thresholds for hypoglycaemia in newborns, was considered for inclusion. Qualitative literature, literature not published in English, and grey literature were excluded for lack of applicability. Papers were also excluded if the participants did not fit the identified population, which was limited to full term, well newborns who were asymptomatic of hypoglycaemia in their first 24 hours of extrauterine life. The PRISMA Extension for Narrative Reviews (PRISMA-ScR) was utilised for reporting due to the applicability to the search and outcomes.

### Data Collection

Five databases—CINAHL, Medline, Scopus, Web of Science, and CENTRAL—were systematically searched in February 2024 for all published literature to date (from database inception). Examination of the references of included papers were also performed. Searching was limited to peer reviewed literature published in English. Our search strategy was (threshold* OR definition OR “normal value”) AND (neonat* OR baby OR babies OR newborn*) AND (hypoglycN=em* OR “blood glucose” OR “BGL” OR plasma glucose OR blood sugar level*). An additional search of the Cochrane Database using the same parameters was performed in August 2024. This search revealed five reviews that were not appropriate for inclusion in this review based on population (maternal or pre-term). Therefore, they were not considered.

### Measurement

Two categorical definitions of hypoglycaemia (statistical and neurodevelopmental) were identified and used to define the result variables. These were the same variables used by all included studies.

The included articles were published in journals from 1989 to 2023, all relating to Medical fields and published in India (one), the Netherlands (two), New Zealand (three), China (one), Turkey (one), Denmark (one), the United States (one), and Israel (two).

### Demographic Information of Journals

Tables were used to organise the data for comparison. Due to the lack of heterogeneity across all included studies, these tables could not be synthesised for direct comparison across all studies and were grouped by categorial definition of hypoglycaemia.

### Data Analysis

Original studies (including RCTs, quasi RCTs, cohort and observational studies) on treatment thresholds for hypoglycaemia in newborns that met the inclusion criteria were included for review. Database search results were uploaded to Covidence software, and all duplicates removed before review for inclusion by two researchers (KH and ST). The first round of screening was conducted independently by title and abstract. Then independent full article screening was conducted to assess appropriateness for inclusion against the identified criteria. Independent results were not unified until extraction was complete. All decisions were made unanimously. Reasons for exclusion in full-text analysis were documented. Reference lists of all full studies were checked for further studies for inclusion. One was considered appropriate. A data extraction table was established and utilised to independently document the data, including study design and methodology (Tables 3 and 4). Studies whose data could be utilised to produce a statistical definition were grouped together and sorted according to the hours of life they tested, noting sample size, to help decipher trends in data.

For neurodevelopmental findings, we reported the age that assessment was attended, the assessment tools used, the different results the two comparison groups received, the identified treatment threshold, and whether the participants experienced a hypoglycaemic episode.

Data extraction was performed by two researchers (KH and ST) independently and collated. Missing data from studies is acknowledged in the discussion. The methodological results and quality of each study could not be synthesised by grouping similar results to identify trends. The results have been grouped by hypoglycaemic definition as summarised below.

### Study Selection

**Figure 1.**
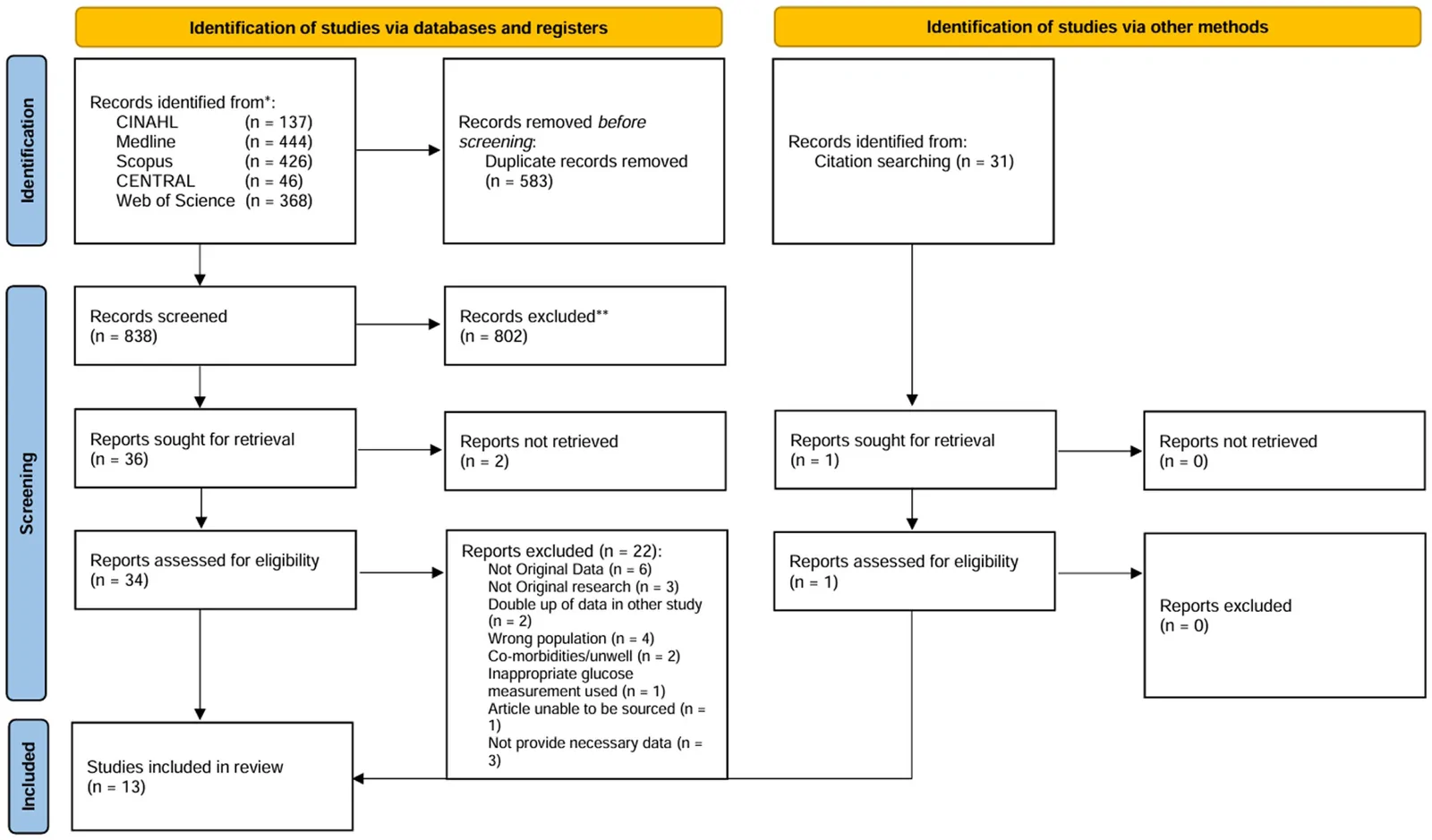
Prisma flowchart.

The initial search yielded 1421 results following the automatic removal of 583 duplicates (see Figure 1: Prisma Flowchart). Following title and abstract screening, 802 records were excluded. In total, 36 studies were sought for retrieval, with 35 papers received. One paper was not able to be retrieved due to lack of availability in English. One further study was identified by searching the references of the papers cited. After reading the full text papers, 13 met the inclusion criteria and are included in this review.

## Results

### Risk of Bias Within Studies

The Crowe Critical Appraisal Tool (Version 1.4; Crowe, 2013) was used to assess the quality of the design, sampling, data collection, ethical matters, results, and discussion for each study. This tool was used to determine the strength and quality of the findings of each study. Two researchers (KH and ST) performed the quality assessment independently and results were averaged to whole numbers for inclusion (see Table 1). No conflicts were identified for discussion and agreement on analysis with the author group.

Supplemental Table 3 outlines the quality assessment undertaken. Two studies were assessed as having a “very high risk” of bias, six as “moderate risk” and five as “low risk.” The two studies assessed as having a “very high risk of bias” (Bhalla et al., 1978; Koh et al., 1988) were excluded from further analysis due to their poor rating and identified unreliability of results.

### Study Characteristics

Tables 1 and 2 summarise the characteristics of the 13 included papers. Studies were conducted in India (one), the Netherlands (two), New Zealand (three), China (one), Turkey (one), Denmark (one), the United States (one), and Israel (two). Data collected included point-of-care testing (POCT; Harris et al., 2020; Levy-Khademi et al., 2019), blood gas analysis (McKinlay et al., 2015), formal laboratory pathology (Brand et al., 2005; Diwakar & Sasidhar, 2002; Koh et al., 1988), outdated technology (Bhalla et al., 1978; Heck & Erenberg, 1987; Koh et al., 1988), and unspecified testing methods (Rasmussen et al.). The population sample sizes ranged from *n* = 17 to *n* = 3595. Both observational (e.g., cohort studies) and experimental studies (e.g., RCTs) were included.

**Table 1.** Summary of Included Papers.

Authors	Aim/Research Question	Methodology	Participants
Harris et al. (2020)	“To determine postnatal changes in plasma and interstitial glucose concentrations of healthy infants receiving current recommended care and to compare the incidence of low concentrations with recommended thresholds for treatment of at-risk infants.”	Prospective masked observational cohort study.	*N* = 67
Rasmussen et al. (2020)	“We performed a follow-up of a cohort of neonates with blood glucose recordings < 1.7 mmol/L, treated with > 2.5 mmol/L, compared with healthy siblings.”	Observational follow-up study.	*N* = 103 (36 girls/45 boys)
Van Kempen et al. (2020)	“We sought to determine whether a management strategy that used a lower threshold . . . 2.6 mmol/L . . . would be noninferior to a traditional threshold . . . 2.6 mmol/L . . . with respect to psychomotor development at 18 months.”	Multicenter, randomised, controlled, noninferior trial.	*N* = 689/582*
Bromiker et al. (2019)	“To determine the true incidence of early neonatal hypoglycaemia and to confirm potential risk factors.”	Cohort study using universal point of care screening.	*N* = 3595
Levy-Khademi et al. (2019)	“There is no single initial value of glucose that can be used with certainty of safety” so “We sought to characterize the normal values of blood glucose levels in a large cohort of neonates.”	The data of all babies admitted to the Well-Baby Nursery from June-Sep 2014 had their data extracted post D/C.	*N* = 3, 912
Qiao et al. (2019)	To follow up the “neurodevelopment in 2 year old infants who had suffered from neonatal hypoglycaemia.”	Retrospective Cohort Study.	*N* = 195 + 144 control
McKinlay et al. (2015)	To investigate “the relation between duration, frequency, and severity of low glucose concentrations in the neonatal period and the neurophysiological development at 2 years.”	Prospective Cohort Study.	*N* = 528/404*
Kayiran and Gürakan (2010)	“To screen BGLs in healthy term and near-term neonates, and to assess the influence of mode of delivery, birth weight and gestational age on BGLs.”	Retrospective cohort study of healthy term and near-term neonates in the first hour of life from clinical charts.	*N* = 1540
Brand et al. (2005)	“To evaluate the effects of transient hypoglycaemia on the first day of life in 75 healthy term large for gestational age infants, born to non-diabetic mothers, on their neurodevelopmental outcome at the age of 4 years.”	Tested 75 x 4-year olds’ neurodevelopmental function, identified from clinical notes audit from their year of birth.	*N* = 75
Diwakar and Sasidhar (2002)	To assess the influence the following has on neonatal glucose levels; maternal parity, mode of delivery, time since last feed, length of intervals between feeds and age of baby (in hours).	Longitudinal Cohort study.	*N* = 200
Koh et al. (1988)	Measure “neural function in relation to blood glucose concentrations” and “to determine if there is a measurable difference in neural function between children who are ‘symptomatic’ and ‘asymptomatic’ during periods of low blood glucose concentrations.”	“We measured the sensory evoked potentials in relation to blood glucose concentration in 17 children.”	*N* = 17 (only two are included in this review as only two of 17 fit our inclusion criteria)
Heck and Erenberg (1987)	“To define normal values of serum glucose levels during the first 48 hours of life in well term neonates” and compare those breastfed with those bottle fed.	Pregnant woman fitting the studies criteria were enrolled in the study and subsequently their newborns blood glucose levels were monitored for the first 48 hr of life.	*N* = 114
Bhalla et al. (1978)	“Norms relating to Indian newborns regarding blood glucose levels are not satisfactorily available. Therefore, it was decided to study blood glucose levels during the early neonatal period.”	Monitored the blood glucose levels of well newborns for the first 168 hr of life.	*N* = unknown (min 30)^a^

* X/Y, where X= N= of participants receiving glucose monitoring at birth and Y= N= of participants who presented for their neurodevelopment assessments later in life.

a Bhalla and colleagues’ (1978) paper fails to state the exact *N* of participants in the study; the results table indicates there were at least 30 participants.

**Table 2.** Summary of Interventions in Included Papers.

Authors	Interventions/Glucose Monitoring	Authors Conclusion	Perceived Limitations
Harris et al. (2020)	Interventions: Monitored neonates’ BGLs, via two different techniques. BGLs checked pre-feeds where possible Glucose monitoring: (1) mmol/L; (2) POC and blood gas machine analysis; (3) first sample within first 4 hr of life, then repeated every 3–4 hr for first 24 hr, then every 12 hr until 5 days old; (4) Yes.	More frequent and severe hypos were detected though interstitial than intermittent testing. “Individual infants appeared to take varying periods of time to stabilise” Babies born at < 40 weeks gestation were more likely to have hypo.	Small sample size. POC monitor used.
Rasmussen et al. (2020)	Interventions: “Evaluate the impact of transient moderate or severe neonatal hypo with a longer follow up to 6–9 years of age with determination of cognitive, motor and behaviour scores. Comparison to healthy siblings as controls were used. Glucose monitoring: (1) mg/dl; (2) unidentified; (3) unidentified; (4) yes.	“In neonates without other severe factors for neurodevelopmental impairment but hypo < 1.7 mmol/L treated with > 2.5 mmol/L, we only detected a lower fine mother function within the normal range at follow up, especially in boys. No changes in cognitive function or behaviour were found.”	Unidentified timing of BGL checks. Unidentified BGL measurement technique. Siblings were considered control group.
Van Kempen et al. (2020)	Interventions: Neonates in the trial were split into two groups; Group 1 were cared for with the treatment threshold of 2.6 mmol/L/ Group 2 were cared for with the treatment threshold of 2.0 mmol/L. Neurodevelopmental assessment at 18 months old. Glucose monitoring: (1) mmol/L and mg/dl; (2) not specified; (3) not specified; (4) yes.	Supports the treatment threshold of 2.0 mmol/L. This recommendation made off their findings that treating at 2.0 mmol/L caused nil increased incidence of neurodevelopmental issues at 18 months when compared to treating at 2.6 mmol/L.	Did not consider other causes of neurodevelopmental abnormalities when analyzing results. Inconsistent testing times and methods; different participating hospitals (17) potentially used different methods. Neurodevelopmental function was assessed at 18 months, which is not a good indicator of future function.
Bromiker et al. (2019)	Interventions: All babies admitted underwent 2 hr observation during transition period with BGL screening before oral feedings. Glucose monitoring: (1) mg/dl; (2) POC; (3) M 74 min +/- 30 min; (4) yes.	3.4% had BGL < 40mg/dl and 12.1% had BGL < 47mg/dl. Gestational age (strongest factor), maternal diabetes, low birth weight (< 2500 g) and twin delivery associated with early neonatal hypo. Mean BGL increases with gestation.	Testing did not continue after feeding. POC monitor used.
Levy-Khademi et al. (2019)	Interventions: Every baby admitted to the Well-Baby Nursery during study had their glucose level checked. Glucose monitoring: (1) Mg/dl; (2) POC; (3) Within the first 180 min of life (M 73 min); (4) yes.	Only 1.5% of participants had a reading below 2.0 mmol/L (converted mg/dl). Nil statistical significance difference in BGL mean between different sexes, weight for age or mode of birth. Mean BGL increases with gestation.	Nil exclusion criteria, and inclusion criteria non-specific Not documented which of the participants had/had not had first feed before glucose test POC monitor used.
Qiao et al. (2019)	Interventions: Monitored neonates’ BGLs of mothers who had gestational diabetes within 0.5 hr after birth. Followed up with Neurodevelopmental assessment at 2 years of age. Glucose monitoring: (1) mmol/L; (2) blood gas machine analysis; (3) < 2 hr/2–24 hr/ > 24 hr; (4) yes.	Found no significant difference in any assessment score of neurodevelopment between Group A and control group. Babies born to mothers with increased weight gain or using insulin in perinatal period were prone to long and repeated neonatal hypo.	Did not consider other causes of neurodevelopmental causes when analysing results. POC monitor used. Neurodevelopmental function was assessed at 2 years; not a good indicator of future function.
McKinlay et al. (2015)	Interventions: Monitored neonates’ BGLs. Neurodevelopmental assessment at 2 years of age. Glucose monitoring: (1) mg/dl; (2) blood gas machine analysis; (3) “regularly” for first 48 hr of life but nil specific schedule documented; (4) yes.	Recommends the use of 2.6 mmol/L as the optimum BGL treatment threshold. Recommendation made off their findings that treating to maintain a BGL > 2.6 mmol/L resulted in nil increased incidence of neurodevelopmental or processing difficulty.	Did not consider other causes of neurodevelopmental causes when analysing results. Inconsistent timing of BGL checks. BGL measurement technique not specified. 237 of the neonates in this study were simultaneously part of another trial. Neurodevelopmental function was assessed at 2 years; not a good indicator of future function.
Kayiran and Gürakan (2010)	Interventions: Healthy on appearance, born at or near-term gestation, asymptomatic babies being breastfeed were tested using POC. Glucose monitoring: (1) mg/dl; (2) POC; (3) 30–60 min; (4) yes.	Vaginal births resulted in higher neonatal BGLs. Higher BGL significantly correlated with higher gestational age. “Screening asymptomatic, healthy term and near-term neonates for hypo in the first hour following birth is unnecessary.”	Near-term participants excluded from our review. POC monitor used.
Brand et al. (2005)	Interventions: Neurodevelopmental assessment at 4 years of age. Glucose monitoring: (1) Mmol/L; (2) formal laboratory chemistry; (3) At 1, 3 and 5 hr of life; (4) yes.	After assessing the neurodevelopmental assessment results utilising three different hypo parameters, they concluded that there is no statistically significant difference in function between those who did and did not experience a hypo. Recommend “at risk” babies don’t need BGL monitoring except SGA babies who they found to be at higher risk.	Only 64% of eligible population had neurodevelopmental assessment attended, so “the possibility of selection bias should be considered.” Did not consider other causes of neurodevelopmental abnormalities in first 4 years of life when analysing results. The DDS tool used in this study is inferior to other tools (e.g., BSID).
Diwakar and Sasidhar (2002)	Interventions: Monitored neonates’ BGLs. Glucose monitoring: (1) Mmol/L; (2) formal laboratory chemistry; (3) at 3, 6, 24, and 72 hr of life; (4) yes.	“We believe that, in the absence of risk factors, it is best to leave the term, breast feeding infant alone and not embark on treatments based on biochemically determined values.” Parity, mode of birth, and time since last feed all had nil effect on BGL, although how many hours old the baby was had some.	Information on why sample size was chosen and how recruitment occurred were not provided.
Koh et al. (1988)	Interventions: Monitored children’s glucose levels while monitoring brainstem auditory evoked potentials. Some participants had hypo induced/other spontaneous. Glucose monitoring: (1) Mmol/L; 2) POC testing and an unspecified formal laboratory test which utilised glucose oxidisation; (3) simultaneously to neurophysiological assessment; 4) yes.	Recommend a treatment threshold of 2.6 mmol/L for children. State they have disproven the theory that asymptomatic hypo is less dangerous than a symptomatic one.	Modern technology provides more accurate BGL results, decreasing the applicability of these results produced by older technology. Extremely small sample size. Inconsistent methodology/testing/monitoring.
Heck and Erenberg (1987)	Interventions: Monitored neonates’ BGLs. Glucose monitoring: (1) Mg/dl; (2) POC; (3) at 1 and 2 hr of life, and then 3–4 hr post feeds until 48 hr of life; 4) yes.	Recommend a treatment threshold of 1.7 mmol/L for the first 24 hr of life and 2.2 mmol/L for the second 24 hr of life.	Modern technology provides more accurate BGL results, decreasing the applicability of these results produced by older technology.
Bhalla et al. (1978)	Interventions: Monitored neonates’ BGLs. Rigid feeding schedule, to try and induce hypo. Neonatal temp maintained between 36.1 ºC–36.7 ºC. Glucose monitoring: (1) Mg %; (2) colorimetric method; (3) ≤ 15 min, ½–6 hr, 7–12 hr, 13–18 hr, 19–24 hr and 10 x more frames up to 168 hr old; (4) not specified.	-SGA babies are more likely to experience hypo, and are at higher risk for longer, than their “normal” counterparts. There is a correlation between gestational age and *M* BGL.	*N* = of participants never documented. Unit of measure not convertible to mmol/L. Modern technology provides more accurate BGL results, decreasing the applicability of these results produced by older technology.

*Note.* AAP = American Academy of Pediatrics; AGA = Average for gestational age; BGL = Blood glucose level; Hypo = hypoglycaemia; LGA = Large for gestational age; POC = Point of Care. (

1) = unit of measure; (2) = technique used to measure glucose level; (3) = hours when BGL checked; (4) = if episodes of hypoglycaemia, as per the individual studies’ definition, was treated.

Six studies (Bhalla et al., 1978; Bromiker et al., 2019; Diwakar & Sasidhar, 2002; Heck & Erenberg, 1987; Kayiran & Gürakan, 2010; Levy-Khademi et al., 2019) analysed statistical hypoglycaemic thresholds and five (Brand et al., 2005; Qiao et al., 2019; Rasmussen et al., 2020; Van Kempen et al., 2020) studied neurodevelopmental outcomes of hypoglycaemia in newborns.

The findings of the review are summarised according to these studies’ hypoglycaemic definitions; Table 3 outlines the statistical results and Table 4 outlines the neurodevelopmental results. Table 3 summarises the reference interval lower limits for the varied testing timeframes in an attempt to identify a treatment threshold for each time period in the first 24 hours of life. Table 4 summarises the neurodevelopmental function testing to identify outcomes from those newborns who experienced hypoglycaemic events compared to those who did not.

**Table 3.** Statistical Results.

Author	Age at Which Study Checked Neonatal BGL (as Hours or Minutes of Life), Grouped by Range							
	0–4 Hr		0–12 Hr		4–12 Hr		12–24 Hr	
	Age Study Checked BGL	Reference Interval Lower Limit (mmol/L)	Age Study Checked BGL	Reference Interval Lower Limit (mmol/L)	Age Study Checked BGL.	Reference Interval Lower Limit (mmol/L)	Age Study Checked BGL	Reference Interval Lower Limit (mmol/L)
Harris et al. (2020)	0–240 min (0–4 hr)	1.80	0–12 hr	2.00	4–12 hr	2.00	12–24 hr	2.30
Bromiker et al. (2019)	≤ 180 min (≤ 3hr)	1.83	≤ 3 hr	1.83	-	-	-	-
Levy-Khademi et al. (2019)	≤ 180 min (≤ 3 hr)	2.09*	≤ 3 hr	2.09*	-	-	-	-
McKinlay et al. (2015)	-	-	< 12 hr	2.10	-	-	12–24 hr	2.20
Kayiran and Gürakan (2010)	≤ 60 min (≤ 1hr)	Birth weight. 2.5–3 kg = 1.38 3–3.5 kg = 1.50 3.5–4kg = 1.45 Gestation. 38–39 = 1.47 40+ = 1.69	≤ 1hr	Birth weight. 2.5–3 kg = 1.38 3–3.5 kg = 1.50 3.5–4 kg = 1.45 Gestation. 38–39 = 1.47 40+ = 1.69	-	-	-	-
Brand et al. (2005)	60 min (1 hr)	0.28	1 hr	0.28	5 hr	1.21	-	-
	180 min (3 hr)	1.33	3 hr	1.33	-	-	-	-
	-	-	5 hr	1.21	-	-	-	-
Diwakar and Sasidhar 2002	150–210 min (3 hr +/- 30 min)	0.90	3 hr +/- 30 min	0.90	6 hr +/- 30 min	1.45	24 hr +/- 30 min	1.31
	-	-	6 hr +/- 30 min	1.45	-	-	-	-
Heck and Erenberg (1987)	50–70 min (1 hr +/- 10 min)	1.33	1 hr +/- 10 min	1.33	5–6 hr	1.89	-	-
	110–130 min (2 hr +/- 10 min)	1.72	2 hr +/- 10 min	1.72	-	-	-	-
	-	-	5–6 hr	1.89	-	-	-	-

* Reference interval lower limit was calculated by estimating the 2.5^th^ percentile from the other percentiles provided, instead of by utilising the standard deviation and mean (as was done for the other studies) as they were not provided in this article.

- No data published for this time frame.

**Table 4.** Neurodevelopmental Results.

Author	Age of Assessment	Description of Groups		Assessment Tool	Mean Score of Groups		Did Author(s) Declare a Statistically Significant Difference?
		Group A: Experimental Group	Group B: Control Group		Group A	Group B	
Rasmussen et al. (2020)	7.75 years (median age)	Experienced moderate or severe neonatal hypo. Moderate = 0.5–1.0 mmol/L at < 2 hr old & 1.0–1.6 mmol/L at > 2 hr old. Severe = < 0.5 mmol/L at < 2hr old & < 1.0 mmol/L at > 2hr old	Siblings of children in group A who themselves never had neonatal hypo.	WISC-IV, Total score. (Cognitive function)	Overall = 96.8 Mod = 96.4 Severe = 99.0	99.7	No
				Movement ABC-2, Total motor. (Motor function)	Overall = 48.3 Moderate = 48.0 Severe = 49.2	60.7	Yes
				Movement ABC-2, Fine motor. (Motor function)	Overall = 42.6 Moderate = 43.3 Severe = 40.4	57.2	Yes
				CBCL, Internalizing. (Behaviour)	Overall = 54.1 Moderate = 62.4 Severe = 56.2	61.5	No
				CBCL, Externalising. (Behaviour)	Overall = 52.2 Moderate = 53.1 Severe = 42.1	50.1	No
Van Kempen et al. (2020)	18 months^	Lower threshold. Received treatment at BGL 2.0 mmol/L.	Traditional threshold. Received treatment at BGL 2.6 mmol/L.	Bayley-III-NL	Cognitive = 102.9 Motor = 105.6	Cognitive = 102.2 Motor = 104.9	No
Qiao et al. (2019)	2 years old^	Experienced neonatal hypo, defined as < 2.6mmol/L. Group A1 = Hypo was rectified, and did not re-occur, by 2 hr of life. Group A2 = Hypo was not rectified until 2–24 hr or a repeat hypo happened in this time.	Infants whose mother did not have GDM and who did not have a hypo.	GESELL, Gross motor	A1= 87.4 A2 = 84.6	86.1	No
				GESELL, Finer motor	A1 = 90.2 A2 = 86.8	91.8	No
				GESELL, Language	A1= 91.7 A2= 88.5	89.6	No
				GESELL, Adaptability	A1= 83.6 A2= 73.9	87.9	No, for A1 vs. B Yes, for A2 vs. B
				GESELL, Social skills	A1= 84.4 A2= 82.6	84.8	No
McKinlay et al. (2015)	2 years^	Neonates who had ≥ 1 hypo. Study defined hypo as a BGL < 2.6 mmol/L.	Neonates who had no hypo. Study defined hypo as a BGL < 2.6 mmol/L.	BSID-III	Cognitive = 93.8 Language = 95.5 Motor = 98.6 Social Emotional =104.3 Adaptive Behaviour = 99.6 Developmental Delay = 70 (30)*	Cognitive = 93.1 Language = 93.8 Motor = 98.7 Social/Emotional = 100.5 Adaptive Behaviour = 98.4 Developmental Delay = 68 (36)*	No
				Executive function test	10.6	10.3	No
				Global motion perception test	Motion coherence threshold = 41.5	Motion coherence threshold = 43.2	No
				BRIEF-P	Global T score > 65 = 46 (21)*	Global T score > 65 = 44 (24)*	No
				Visual & Audiologic screening, and carer completed questionnaires on the child’s health and home environment.	No raw data published for these assessments.	No raw data published for these assessments.	No, for any
Brand et al. (2005)	4 years old	Neonates who had ≥ 1 hypo. Study defined hypo as a BGL < 2.2 mmol/L at ≤ 1hr old and < 2.5 mmol/L thereafter.	Neonates who had no hypo. Study defined hypo as a BGL < 2.2 mmol/L at ≤ 1hr old and < 2.5 mmol/L thereafter.	DDS	93% ~	100%~	No
				SON IQ	108.9	114.3	No
				CBCL	51.3	52.9	No

*Note.* BGL = Blood Glucose Level; Hypo = Hypoglycaemia; WISC-IV= Wechsler’s intelligence scale for children fourth edition; Movement ABC-2= Movement Assessment Battery for Children 2; CBCL= Achenbach Child Behaviour Checklist; Bayley-III-NL = Bayley Scales of Infant and Toddler Development, 3rd edition, Dutch version; GDM= Gestational diabetes mellitus; GESELL= Gesell Infant Development Scale (Chinese revised edition); BSID-III = Bayley Scales of Infant Development III; BRIEF-P = Behaviour rating Inventory of Executive Function-preschool version; DDS = Denver Developmental Scale, Dutch version; SON-IQ = Snijders-Oomen non-verbal intelligence test; CBCL = Child Behaviours Check List, Dutch version.

^ = age corrected for prematurity where appropriate.

* = as *N* = of participants and % of participants.

~ = % of participants scores that were classified as “definitively normal.”

BGLs that were produced by techniques other than blood sampling from the newborn were not included due to their inconsistency, such as samples taken “at birth” from umbilical cords (Heck & Erenberg, 1987), and interstitial results (Harris et al., 2020; McKinlay et al., 2015). All conversions of mg/dl to mmol/L were performed with the understanding that a mmol weighs 180.2 mg, and 10 dl being in 1 L, with the results of the equation being rounded to 2 decimal points.

### Statistical Outcomes

Six studies (Bhalla et al., 1978; Bromiker et al., 2019; Diwakar & Sasidhar, 2002; Heck & Erenberg, 1987; Kayiran & Gürakan, 2010; Levy-Khademi et al., 2019) investigated the statistical BGL thresholds in newborns. All studies utilised either point of care testing (POCT) using machines and oxidised strip testing, blood gas analysis or formal laboratory testing. All these methods are noted to have different accuracy. All studies utilised different time periods of testing, with some testing directly at birth and others waiting until the newborn had completed a feed. Our review looked exclusively at the testing in the first 24 hours of extrauterine life, so data from other time periods were not reported.

The range of statistical BGL reference interval lower limits for hypoglycaemia in newborns in the first 12 hours of extrauterine life was 0.28 mmol/L ( to 2.10 mmol/L (McKinlay et al., 2015). The reference interval lower limit at 0–4 hours was 0.28 mmol/L (Brand et al., 2005) to 2.09 mmol/L (Levy-Khademi et al., 2019). Specifically, statistical BGLs identified in the first 4 hours of extrauterine life confirmed the presence of a physiological nadir that occurs as the known adaption to extrauterine life occurs (Adamkin, 2017; Cornblath et al., 2000).

The reference lower interval for the 12–24 hours of life was 1.31 mmol/L (Diwakar & Sasidhar, 2002) to 2.3 mmol/L (Harris et al., 2020). Based on the largest data set, with lowest risk of bias, the lower limit for normal glucose in newborns was identified as 2.09 mmol/L for the first 3 hours of life (Levy-Khademi et al., 2019).

### Neurodevelopmental Outcomes

Five studies (Brand et al., 2005; McKinlay et al., 2015; Qiao et al., 2019; Rasmussen et al., 2020; Van Kempen et al., 2020) analysed the neurodevelopmental outcomes of newborns who had either experienced hypoglycaemia or were at risk of experiencing hypoglycaemia after birth. Testing was performed utilising POC machines (Brand et al., 2005; McKinlay et al., 2015; Van Kempen et al., 2020) and interstitial monitoring (Van Kempen et al., 2020).

All five studies found that there was no statistically significant difference between the scores of the neurodevelopmental outcomes. In fact, the newborns who experienced hypoglycaemia were more likely to have better outcomes in the Child Behaviour Checklist (CBCL) T score (Brand et al., 2005), motion coherence, developmental delay, Global T scores, social and emotional development (McKinlay et al., 2015), and a reduced occurrence of seizures (McKinlay et al., 2015). Males were found to have lower fine motor scores within the normal range compared to females (Rasmussen et al., 2020). There were no reported increased risks of neurodevelopmental injury identified with an increased number or severity of hypoglycaemic episodes (McKinlay et al., 2015). Of further note, those newborns who experienced untreated asymptomatic hypoglycaemic episodes that were retrospectively identified from interstitial testing, were not found to have any increased incidence of neurodevelopmental delay or injury (Van Kempen et al., 2020).

McKinlay et al. (2015) incidentally found that newborns who had received treatment with (oral or intravenous) glucose in the first 24 hours that caused a rapid increase in their BGLs to the accepted threshold were in the group that experienced higher rates of rebound hypoglycaemia and increased rates of poorer outcomes in general developmental neurological outcomes. This suggests that further investigation is required to ensure that treatment is not causing harm.

Overall, the studies (Brand et al., 2005; McKinlay et al., 2015; Qiao et al., 2019; Rasmussen et al., 2020; Van Kempen et al., 2020) found that there were no statistically significant differences in outcomes between BGL treatment thresholds of 2.0 mml/L versus 2.6 mmol/L, or between participants who experienced a hypoglycaemic episode and those who did not. Whilst these studies reported that 2.6 mmol/L was a safe threshold (Brand et al., 2005; McKinlay et al., 2015; Qiao et al., 2019; Rasmussen et al., 2020; Van Kempen et al., 2020). Van Kempen et al. (2020) provided evidence for consideration that a lower level, such as 2.0 mmol/L, was also a potentially safe treatment threshold and required further investigation.

## Discussion

The quality of evidence currently informing the recommendations to commence the treatment of hypoglycaemia in the well, asymptomatic, term newborn with (maternal) risk factors in the first 24 hours of life is variable. The included studies provide differing degrees of risk of bias, reliability and outcomes, recommendations, and conclusions. The evidence of a physiological nadir was reaffirmed, which suggests that the timing of testing is important.

The major concern is the quality of evidence available. Three of the five studies that presented a statistical focus (Bhalla et al., 1978; Brand et al., 2005; Diwakar & Sasidhar, 2002; Heck & Erenberg, 1987) were found to have a significant risk of bias with very small sample sizes and low study quality.

When reporting the neurodevelopmental outcomes, no statistically significant differences were found between those newborns who experienced at least one hypoglycaemic event (with thresholds of between 2.0 mmol/L and 2.6 mmol/L) and those who did not. Whilst there were some similarities in the testing methods between the studies, there were several issues identified with these methods. The Bayley-III testing methods have been found to be unreliable in determining the continuance of neurodevelopmental delay when used at 2 years and 4.5 years (Burakevych et al., 2017). Although the BSID-III has some limitations, it is considered a good assessment tool and superior to the Denver Developmental Scale (DDS) tool. The DDS tool was used by Brand and colleagues (Brand et al., 2005), who acknowledged this fact. An uncommonly used Gesell Infant Development Scale was utilised by Qaio and colleagues (Qiao et al., 2019) and a combination of the Wechsler IV cognitive test, Movement ABC-2 test and Child Behaviour Checklist were used in Helleskov Rasmussen and colleagues’ work (Rasmussen et al., 2020).

The current testing methods do not recognise the potential for other factors to affect the neurodevelopment of the participants, thereby potentially invalidating the results. There was no clarification or recognition of the rate of neurodevelopment injury in the well population of newborns overall (Cha et al., 2022; Chung et al., 2020) or acknowledgement of the potential for other participant characteristics or health events to have occurred prior to or since the hypoglycaemic events. Both factors may have affected neurodevelopmental outcomes at the testing points of 18 months, and 2 and 4 years (Cha et al., 2022; Chung et al., 2020). Despite late pre-term newborns being included in some studies, their statistically increased rate of neurodevelopmental injury or delay due to being preterm was not acknowledged (Cha et al., 2022; Chung et al., 2020).

Overall, the quality of most studies included in the systematic review was poor. The risk of bias was significant for most (Bhalla et al., 1978; Brand et al., 2005; Diwakar & Sasidhar, 2002; Heck & Erenberg, 1987; Koh et al., 1988). The sample sizes were statistically insignificant for all but five (Bromiker et al., 2019; Kayiran & Gürakan, 2010; Levy-Khademi et al., 2019; McKinlay et al., 2015; Van Kempen et al., 2020).

A discussion of one of the studies that was excluded from analysis is warranted (Koh et al., 1988). Exclusion was due to its very high risk of bias, with the lowest and most statistically insignificant sample size. Despite this limitation, it is one of the most highly cited neonatal hypoglycaemia studies (> 250 citations; BMJ, 2024) and is included in many significant policies for hypoglycaemic testing in newborns (ACT Health, 2021; De Angelis et al., 2021; Harris et al., 2014; Jesitus et al., 2016; National Institute for Health and Care Excellence, 2020; New South Wales Government, 2021; The Royal Children’s Hospital Melbourne, 2019; WHO, 2020; UNICEF–UK, 2013). This was the only study that discussed neurophysiological outcomes, reporting that abnormal neurological function was detected in participants in the first day of life, when BGL readings reached a low of 1.9 mmol/L. The authors hypothesised that the BGL threshold of 2.6 mmol/L (the highest end of the operational neonatal hypoglycaemia threshold) should be utilised to provide neural protection for children. Of note, this study had a very small sample size (*n* =17) and included only two neonates in the first 24 hours of extrauterine life (Koh et al., 1988). Due to the outlined characteristics of this study, limited sample sizing, inconsistent testing methodology, and lacking strength in outcomes or conclusions, it may be a cause for concern that these findings continue to inform policy and practice.

With regards to the timing of testing, evidence was identified reaffirming the existence of a physiological nadir in BGLs in newborns, the lowest point at approximately 2 hours post birth and rising again by 3 to 4 hours. Normal glycaemic levels are not expected to be reached until 72–96 hours of age. However, most current policies recommend testing BGLs in asymptomatic newborns in the first 1–2 hours of life (ACT Health, 2021; De Angelis et al., 2021; Harris et al., 2014; Jesitus et al., 2016; National Institute for Health and Care Excellence, 2020; New South Wales Government, 2021; The Royal Children’s Hospital Melbourne, 2019; UNICEF–UK, 2013; WHO, 1997). The data presented in some of the studies makes it difficult to identify if the BGL resolved without intervention. A recent study (Harris et al., 2020) identified (with interstitial monitoring) the well term asymptomatic newborn’s innate ability to resolve their hypoglycaemia without intervention. This suggests that intervention should be carefully considered for equity with risks.

Human milk has been shown to maintain adequate BGLs in most newborns and continues to provide adequate nutrition to support optimal growth and development (Cordero et al., 2018; Dalsgaard et al., 2019; World Health Organization, 2003). Research reveals that unrestricted effective breastfeeding engages the majority of the newborns’ own physiological processes and ensures that their metabolic function is appropriate for the hours of life (Cordero et al., 2018; Dalsgaard et al., 2019; World Health Organization, 2003). We could not identify any studies that demonstrated that an asymptomatic hypoglycaemic event within the nadir period causes any short or long-term morbidity or mortality (Deshpande & Platt, 2005; National Institute for Health and Care Excellence, 2020; Rozance & Hay, 2010).

The studies included in this systematic review have revealed that an inconsistency exists in hypoglycaemia management. The evidence provided by these studies does not appear to robustly support using a BGL of 2.6 mmol/L as a treatment threshold for well term asymptomatic newborns in the first 24 hours of life. This level may be too high and contributing to unnecessary interventions, particularly the use of commercial milk formula, which has numerous well-documented adverse health outcomes (Chantry et al., 2014; Cummins et al., 2020; Mukhopadhyay et al., 2020). High level multidisciplinary discussion that considers whether the risk benefit ratio of intervening at the higher BGL threshold is equitable is warranted. It is outside the scope of this review to suggest that thresholds should be changed. This paper proposes, rather, to act as a conversation starter for midwives and health professionals to consider what evidence is required to unequivocally support the thresholds and guide clinical practice. To support such a discussion consideration for a robust multi-centre, multi-trial, randomised control trial, with consistent parameters for method and timing of testing, to establish a consistent, evidence-based hypoglycaemia treatment threshold in newborns, is suggested.

### Strengths and Limitations

This topic suited a narrative review to integrate existing information and provide data for future decision-making. The heterogeneity did not facilitate meta-analysis and direct data comparison due to the different testing types and times, study sizes, and reporting methods. No direct comparisons can be drawn between the included studies; however, consistent conclusions drawn from the authors have been reported.

Only papers published in or translated into English were included in the review. The included papers are from a range of locations with populations of varying characteristics, and mostly small sample sizes, so the results may not be generalisable across countries/populations.

We note that all included studies used different testing methods and timing. This process may affect the accuracy and/or relatability of those results to the general newborn population. The contemporary applicability of results is further questioned in the studies conducted before 1990, due to reported use of superseded testing methods (Bhalla et al., 1978; Heck & Erenberg, 1987; Koh et al., 1988).

## Conclusion

Published research cannot provide a strong evidence base to definitively determine a therapeutic treatment threshold for hypoglycaemia in well, term, asymptomatic newborns in the first 24 hours of life. There is evidence to suggest that the most frequently used threshold of 2.6 mmol/L is high, and the current protocols for testing times do not account for the normal BGL nadir that occurs. Supplementation of breastfeeding that is not based on clear clinical reasons disrupts normal physiological and psychological processes by interfering with successful breastfeeding establishment.

Further discussions and studies are urgently required. A robust, multi-centre, multi-trial study is suggested as a means to determine the normal range of BGL levels in newborns and to inform an evidence-based treatment threshold and future policy and practice. Developing and implementing clear and standardised policy will ensure health care practitioners are only performing necessary interventions that do not interfere with the appropriate support of breastfeeding given its known benefits for mothers and babies.

## Supplemental Material

sj-docx-1-jhl-10.1177_08903344261426051 – Supplemental material for The Evidence Informing Hypoglycaemia Treatment Thresholds in Asymptomatic, Term, Well Newborns: A Review of Literature and Critical Appraisal of Methodological Challenges Supplemental material, sj-docx-1-jhl-10.1177_08903344261426051 for The Evidence Informing Hypoglycaemia Treatment Thresholds in Asymptomatic, Term, Well Newborns: A Review of Literature and Critical Appraisal of Methodological Challenges by Kylie Hodges, Samantha Trenaman, Mary Bushell, Maryam Bazargan and Marjorie Atchan in Journal of Human Lactation
