# Supplementary material for: The Evidence Informing Hypoglycaemia Treatment Thresholds in Asymptomatic, Term, Well Newborns: A Review of Literature and Critical Appraisal of Methodological Challenges
Source: J Hum Lact. 2026 Apr 13;42(3):569–84. doi: 10.1177/08903344261426051 (PMC13615315; doi:10.1177/08903344261426051)
Supplement: Supplementary file 1 [file sj-docx-1-jhl-10.1177_08903344261426051.docx]

**Supplementary Table 1 Search example table**

| **Database Searched:** | Medline |
| --- | --- |
| **Date Search Conducted:** | 29^th^ January 2024 |
| **Search terms:** | (threshold* OR definition OR “normal value”)  AND  (neonat* OR baby OR babies OR newborn*)  AND  (hypoglycN=em* OR “blood glucose” OR BGL or “blood sugar”) |
| **Search Inclusions:** | English Language  Peer Reviewed |
| **Search Exclusions:** | NIL |
| **Search Results:** | 444 |

**Supplementary Table 2	Excluded Articles**

| **Article** | **Exclusion Reason(s)** |
| --- | --- |
| Anderson Enni et al. (2023) DOI: [10.1136/bmjpo-2022-001766](https://dx.doi.org/10.1136/bmjpo-2022-001766) | Did not provide necessary data |
| Harris, et al. (2023) DOI: [10.1136/archdischild-2022-324148](https://dx.doi.org/10.1136/archdischild-2022-324148) · Ref ID: 35940872 | Not original research (data) |
| Gupta, K et al. (2022). DOI: [10.1542/peds.2021-050733](https://dx.doi.org/10.1542/peds.2021-050733) | Did not provide necessary data |
| Gray, et al (2021) DOI: [10.1136/archdischild-2020-319938](https://dx.doi.org/10.1136/archdischild-2020-319938) | Not original research (review) |
| McGann et al. (2021) DOI: [10.1038/s41372-021-00921-1](https://dx.doi.org/10.1038/s41372-021-00921-1) | Not original research (review) |
| Pandey, R & Scheid, L.M. (2020) DOI: [10.1111/apa.15314](https://dx.doi.org/10.1111/apa.15314) | Not original research (review) |
| Harris, et al. (2019). DOI: [10.1111/jpc.14409_58](https://dx.doi.org/10.1111/jpc.14409_58) | Abstract (double up of other included data) |
| McKinlay et al. (2017) DOI: [10.1001/jamapediatrics.2017.1579](https://dx.doi.org/10.1001/jamapediatrics.2017.1579) | Not correct population |
| Tan, et al (2017). DOI: [10.1136/bmjpo-2017-000175](https://dx.doi.org/10.1136/bmjpo-2017-000175) | Not correct population |
| Juthani, K & Kumar, S; Williams, AF (2013). DOI: [10.1136/archdischild-2013-304580](https://dx.doi.org/10.1136/archdischild-2013-304580) | Not correct population |
| Bührer, C. (2016). DOI: [10.1007/s11428-015-0045-6](https://dx.doi.org/10.1007/s11428-015-0045-6) | Not original research (data) |
| McKinlay, et al. (2016). DOI: [10.1111/jpc.13194](https://dx.doi.org/10.1111/jpc.13194) | Abstract (double up of other included data) |
| Greenhill, C. (2015). DOI: [10.1038/nrendo.2015.188](https://dx.doi.org/10.1038/nrendo.2015.188) | Not original research (data) |
| HAPO Study Cooperative Res Grp, et al. (2010). DOI: [10.1542/peds.2009-2257](https://dx.doi.org/10.1542/peds.2009-2257) | Did not provide necessary data |
| Van Howe, RS & Storms, MR. (2008) DOI: [10.1055/s-2008-1076602](https://dx.doi.org/10.1055/s-2008-1076602) | Not correct population |
| Alkalay, et al. (2006) DOI: [10.1177/0009922806290610](https://dx.doi.org/10.1177/0009922806290610) | Not original research (data) |
| Thomas et al (2000) DOI: [10.1046/j.1440-1754.2000.00510.x](https://dx.doi.org/10.1046/j.1440-1754.2000.00510.x) | Not original research (data) |
| LeBlanc, et al. (1997). DOI: [10.1159/000244490](https://dx.doi.org/10.1159/000244490) | Not correct population |
| Holtrop, P. C. (1993) DOI: [10.1055/s-2007-994649](https://dx.doi.org/10.1055/s-2007-994649) | Not correct population |
| Fanconi, et al (1982). | Unable to be sourced |
| Hay Jr, W. W. (1979). | Not original research (review) |
| Monteiro, et al. (1974) | Inappropriate glucose measurement used |

**Supplemental Table 3**. **Risk of Bias & Quality Assessment Scores (CCAT)**

| **Authors** | **N=1**  (Preliminaries) | **N=2**  (Introduction) | **N=3**  (Design) | **N=4**  (Sampling) | **N=5**  (Data collection) | **N=6**  (Ethical matters) | **N=7**  (Results) | **N=8**  (Discussion) | **Total**  (as score/40 & %) |
| --- | --- | --- | --- | --- | --- | --- | --- | --- | --- |
| **Harris et al., 2020** | 5 | 4 | 5 | 4 | 4 | 4 | 4 | 4 | 34  **85%** |
| **Helleskov Rasmussen et al., 2020** | 3 | 4 | 3 | 3 | 4 | 5 | 4 | 4 | 30  **75%** |
| **Van Kempen**  **et al., 2020** | 5 | 5 | 5 | 4 | 5 | 4 | 5 | 4 | 37  **92%** |
| **Bromiker et al., 2019** | 4 | 4 | 4 | 4 | 4 | 2 | 3 | 4 | 29  **772.5%** |
| **Levy-Khademi**  **et al., 2019** | 4 | 4 | 5 | 4 | 4 | 3 | 4 | 4 | 32  **80%** |
| **Qiao et al., 2019** | 5 | 4 | 4 | 4 | 4 | 5 | 4 | 4 | 34  **85%** |
| **McKinlay et al., 2015** | 4 | 5 | 5 | 4 | 5 | 5 | 4 | 4 | 36  **90%** |
| **Kayiran & Gurakan, 2010** | 4 | 4 | 3 | 4 | 3 | 1 | 3 | 2 | 4  **660%** |
| **Brand et al., 2005** | 4 | 4 | 4 | 3 | 4 | 4 | 3 | 4 | 30  **75%** |
| **Diwakar &**  **Sasidhar, 2002** | 4 | 4 | 4 | 3 | 4 | 3 | 3 | 4 | 30  **75%** |
| **Koh et al.,**  **1988** | 3 | 4 | 3 | 1 | 1 | 3 | 3 | 2 | 20  **50%** |
| **Heck &**  **Erenberg, 1987** | 3 | 4 | 4 | 3 | 3 | 3 | 4 | 4 | 28  **70%** |
| **Bhalla et al., 1978** | 2 | 3 | 2 | 1 | 3 | 0 | 2 | 2 | 15  **38%** |

**Table 1 Legend**

- Section scores /5

| 1-2  High risk of bias | 3-4  Moderate risk of bias | 5  Low risk of bias |
| --- | --- | --- |

- Total score as %

| ≤50%  High risk of bias | 51-75%  Moderate risk of bias | 76-100%  Low risk of bias |
| --- | --- | --- |

- Scores calculated by CCAT tool (v1.4)
- All % rounded to the nearest whole number

Acronyms used; CCAT = Crowe Critical Appraisal Tool v1.4
